# Psychosocial impact of human papillomavirus‐related head and neck cancer on patients and their partners: A qualitative interview study

**DOI:** 10.1111/ecc.12999

**Published:** 2019-01-24

**Authors:** Rachael H. Dodd, Alice S. Forster, Laura A. V. Marlow, Jo Waller

**Affiliations:** ^1^ The University of Sydney, Faculty of Medicine and Health School of Public Health Sydney New South Wales Australia; ^2^ Department of Behavioural Science and Health University College London London UK

## Abstract

**Objective:**

Increasing numbers of patients face the psychosocial challenge of a diagnosis of oropharyngeal squamous cell carcinoma (OSCC) caused by human papillomavirus (HPV). We explored the psychosocial impact of an HPV‐OSCC diagnosis for patients and their partners.

**Methods:**

In‐depth interviews were conducted with patients (*n* = 20) and a subset of their partners (*n* = 12), identified through medical records at two UK hospitals. Interviews were recorded, transcribed verbatim and analysed using thematic Framework Analysis.

**Results:**

Only 12/20 patients interviewed (and five partners) were aware of their HPV status and the main analysis focused on this sub‐sample. In discussing the cause of their cancer, patients and their partners talked about not wanting to know; whether they disclosed the cause of their cancer to others; their reactions to being diagnosed with HPV; the prognosis information they were given and the questions they had about HPV. Most concerns were cancer‐related rather than HPV‐related, but some patients (*n* = 3) described feelings of embarrassment and perceived stigma about HPV.

**Conclusion:**

Some patients and partners who are told HPV is the cause of their OSCC have questions about HPV and seek further information. Concerns and uncertainties about the sexually transmitted nature of HPV need to be addressed by health professionals.

## 1 INTRODUCTION

An increasing number of head and neck cancers (HNC) are attributable to human papillomavirus (HPV) (Chaturvedi et al., 2011). The potential for psychosocial distress and fear of recurrence in HNC patients is high (Badr, Gupta, Sikora, & Posner, 2014; Humphris & Ozakinci, 2006), due in part to the potential for disfigurement of the head and neck region, and dysfunction (e.g., problems with swallowing, eating) after treatment (Singer et al., 2012). However, identifying a sexually transmitted infection as a causal factor may lead to additional distress in patients with HPV‐oropharyngeal squamous cell carcinoma (HPV‐OSCC) and their partners (Gold, 2012; Longacre, Ridge, Burtness, Galloway, & Fang, 2012; Shuman & Wolf, 2010). Common patient concerns related to HPV in HNC have been documented to be how, when and why they got their cancer (Fakhry & D'Souza, 2013). The answers to these questions are often complex and, due to a lack of available evidence, sometimes unanswerable (Chu, Genden, Posner, & Sikora, 2013), with implications both for the patient and their past, present or future partners. Partners or carers of HNC patients have informational needs independent of those of the patients and have fears and concerns about HNC and its consequences (Richardson, Morton, & Broadbent, 2015).

As patients diagnosed with HPV‐OSCC are typically younger than OSCC patients caused by other factors, they will live longer with the consequences of treatment (Moschopoulou, Hutchison, Bhui, & Korszun, 2018). HPV‐OSCC patients could also be required to manage family life, while dealing with the challenges associated with the diagnosis and treatment of their cancer (Gold, 2012). As treatment for HNC has been shown to be associated with significant dysfunction and disfigurement (De Boer, McCormick, Pruyn, Ryckman, & Borne, 1999), HPV‐OSCC patients may need to manage these consequences of treatment long into survivorship (Moschopoulou et al., 2018). These patients also seek expedited rehabilitation so that their lives can return to normal as quickly as possible (Dodd, Marlow, & Waller, 2016). The cervical cancer literature has demonstrated that a HPV diagnosis has the potential to cause feelings of stigma and shame in addition to the anxiety and health concerns usually associated with abnormal cervical screening results (McCaffery, Waller, Nazroo, & Wardle, 2006). Sexual relationships may be affected due to concern over the sexual transmission of HPV (Taberna, Inglehart, Pickard, Fakhry, & Agrawal, 2017) and clinicians should be prepared to potentially talk about these concerns (Reich et al., 2016).

The increasing incidence of HPV‐OSCC worldwide highlights the need for research exploring the impact of the diagnosis on patients and their partners to enable us to understand the implications of the diagnosis. This interview study aimed to explore the psychosocial impact of being diagnosed with HPV‐OSCC for both the patient and the patient's partner.

## 2 METHODS

### 2.1 Participants

Eligible patients were identified by medical staff from medical records at two UK hospitals. Patients were eligible if: they had tested positive for HPV and were at least 1‐year post diagnosis of HPV‐OSCC. All patients were eligible to be invited with no limit on time since diagnosis. We first invited patients diagnosed closest to 1 year ago. Partners were eligible if their partner (the patient) met the patient inclusion criteria stated above. All participants had to be able to communicate in English and be able to give informed consent. Ethical approval was granted by South East London NHS Research Ethics Committee (Reference: 15/LO/0803).

### 2.2 Materials

In‐depth interviews followed a topic guide developed using the existing literature on patient experiences of HNC (Lang, France, Williams, Humphris, & Wells, 2013; Rapoport, Kreitler, Chaitchik, Algor, & Weissler, 1993) and previous qualitative work on HPV and cervical cancer (McCaffery et al., 2004; Waller, McCaffery, Nazroo, & Wardle, 2005). The topic guide (Table 1) was piloted with members of a participatory advisory group. The interviews began with an open question about the participant's experience of diagnosis. The rest of the interview was driven by responses to this question, but included questions about symptoms, diagnosis, psychosocial impact and information needs.

**Table 1:** Interview topic guide—patients

*Introduction*
Open with an introduction to self and the study, giving background and aims of the study—explain a university study, not the NHS Tell the participant how long you expect the interview to take (between 30 min to an hour) and explain that the interview will be tape recorded Explain about confidentiality, and the use of the data Explain don't need to talk about anything uncomfortable with and no right/wrong answers Any questions?
*Patient's experience*
Ask about patients experience of being diagnosed—this is an open‐ended question to enable the patient to talk freely about their experiences
Prompts
Symptoms—Symptom recognition and how long before went to a HCP
Disclosing symptoms to anyone
Diagnosis—Who/how/what/when told
Any difficulties/anxieties
Involved in any care/treatment decisions?
Alone or with a partner?
Referred for any support? (counselling needs, QoL, psych treatment)
**IF** HPV mentioned:
What was talked about
How did you feel
**IF** HPV NOT mentioned:
Did the health professional mention HPV at all?
If so, how did you feel
Psychosocial—Initial feelings
Disclosure to partner/others
Reactions of partner/others
Adjustments/coping/impact on daily life (physical, psychological, social)
Anything to help you cope better?
Changes to relationship?
Effect on others
Feelings about the future
Information needs—any research prior to diagnosis
Amount of information received
Lacking any information?
Look elsewhere for information? If so, where, what helpful/unhelpful
Were you given any written information? Need for it?
*Closing the interview*
Any other comments, issues or suggestions that haven't been raised? Thank interviewee Reassure about confidentiality Give debrief, repeating the aims of the study and leaving details of available support Ask if happy to be re‐contacted later in the study

### 2.3 Procedure

Patients were invited to participate through an information pack sent in the post. The information sheet in the pack informed patients that they were being recruited because of their diagnosis with HPV‐related head and neck cancer. Patients were asked to return a short questionnaire, consent form and contact details form using a freepost envelope. A reminder was sent to non‐responders after 3 weeks. Partners were sent an information pack in the post if patients had indicated on their consent form that they were willing for them to be contacted. Signed consent from partners was obtained on the day of the interview. Patients could participate regardless of whether they had a partner/participating partner.

In‐depth interviews were carried out by RD, separately for patients and their partners. Interviews took place face‐to‐face (*n* = 29) or by telephone (*n* = 3) and lasted 26–84 min. They were recorded and transcribed verbatim. Emerging themes from the transcripts were noted simultaneously with carrying out the interviews. Once no new themes had emerged from three consecutive interviews, this indicated that saturation had been achieved. Data collection ceased at this point (Francis et al., 2010). Information about the subsite of patients’ cancer, stage at diagnosis and treatment received was obtained from their medical records.

### 2.4 Analysis

Data were coded thematically using NVivo 11 (QSR, 2015). RD, JW and AF read 10% of the transcripts each (*n* = 3) and developed codes independently. All coding was then discussed and disagreements were resolved before RD coded all of the transcripts. Once all the data had been coded in NVivo, it was summarised in a framework matrix with rows for participants and columns for themes. Framework Analysis (Ritchie & Spencer, 1994) was chosen because it facilitates comparisons both within and between cases (Gale, Heath, Cameron, Rashid, & Redwood, 2013). Patients and partners were all treated as separate participant cases in a single framework. LM read a further 10% of the transcripts and checked her agreement against the coding framework.

## 3 RESULTS

### 3.1 Sample characteristics

Out of 89 patients sent an information pack, 27 returned a consent form (30% response rate). Patients (*n* = 20) and partners (*n* = 12) were interviewed in September/October 2014 and between September and November 2015. Patients average time since diagnosis was 23 months (range 12–53 months). All were diagnosed with a primary tumour in the tonsil (*n* = 14) or base of tongue (*n* = 5) and all except one had late‐stage disease (stage IV A to C). Patients had received surgery alone (*n* = 1), surgery with chemoradiation (*n* = 5), surgery with radiation (*n* = 5) or radiation only (*n* = 8). The medical records for one patient were not obtainable. The majority of patients were male, white British, married or cohabiting, and either employed full‐time or retired (see Table 2).

**Table 2:** Patient and partner characteristics

	Patient characteristics (*n* = 20)	Partner characteristics (*n* = 12)
Age at diagnosis [median (range)]	57 (40–82)	/
Time since diagnosis in months [median (range)]	23 (12–52)	/
Age at interview [median (range)]	59 (41–83)	60 (45–74)
Sex [*n*]		
Male	14	2
Female	6	10
Ethnicity [*n*]		
White British	19	12
Other	1	/
Marital status [*n*]		
Married/Cohabiting	14	12
Widowed	2	/
Divorced	2	/
Separated	1	/
Single	1	/
Employment status [*n*]		
Employed full‐time	7	1
Employed part‐time	2	5
Unemployed	1	/
Retired	8	5
Disabled or too ill to work	2	1
Knew their/partner's cancer was HPV‐positive [*n*]	12	5

Social grade, education and general health characteristics of the recruitment hospitals areas are shown in Table 3 (I Live Here UK, 2018).

**Table 3:** Recruitment hospitals area characteristics (I Live Here UK, 2018)

	Hospital 1 (%)	Hospital 2 (%)
Social grade^a^		
AB	16	28.6
C1	32.4	37.4
C2	16.4	15.1
DE	35.2	18.9
Education^b^		
No qualifications	28.7	16.1
Level 1	12.5	11
Level 2	14.2	12.6
Apprenticeship	2.5	2.1
Level 3	15.2	16
Level 4	22.4	36.9
Other	4.4	5.4
General health		
Very good	46.7	49.3
Good	30.5	33.6
Fair	14	11.8
Bad	6.7	4.1
Very bad	2.1	1.2

a Social grade: AB Higher & intermediate managerial, administrative, professional occupations; C1 Supervisory, clerical & junior managerial, administrative, professional occupations, C2 Skilled manual occupations; DE Semi‐skilled & unskilled manual occupations, Unemployed and lowest grade occupations

b Education: Level 1 (1–4 O Levels/CSE/GCSEs (any grades), Entry Level, Foundation Diploma, NVQ Level 1, Foundation GNVQ, Basic/Essential Skills); Level 2 (5+ O Level (Passes)/CSEs (Grade 1)/GCSEs (Grades A*‐C), School Certificate, 1 A Level/2–3 AS Levels/VCEs, Intermediate/Higher Diploma, Welsh Baccalaureate Intermediate Diploma, NVQ level 2, Intermediate GNVQ, City and Guilds Craft, BTEC First/General Diploma, RSA Diploma; Level 3 (2+ A Levels/VCEs, 4+ AS Levels, Higher School Certificate, Progression/Advanced Diploma, Welsh Baccalaureate Advanced Diploma, NVQ Level 3; Advanced GNVQ, City and Guilds Advanced Craft, ONC, OND, BTEC National, RSA Advanced Diploma); Level 4 (Degree, Higher Degree, NVQ Level 4–5, HNC, HND, RSA Higher Diploma, BTEC Higher level, Foundation degree (NI), Professional qualifications; Other (Vocational/Work‐related Qualifications, Foreign Qualifications (not stated/level unknown).

### 3.2 Overview

Data were examined to understand the psychosocial impact of being diagnosed with HPV‐OSCC, looking at individual experiences for both the patient and their partner, and to explore how this varied between participants. Some initial reactions to being diagnosed with cancer included shock, panic, feeling destined for the diagnosis, thinking they were going to die, and feeling vulnerable and frightened. A small number of patients said the diagnosis was not a shock and a few of them felt like it was not happening to them.

Twelve patients in the sample were aware of the cause of their cancer being HPV, whereas the rest were not. The first theme explores causal beliefs across the whole sample and includes discussion of the information patients received from health professionals about the cause of their cancer. Subsequent themes focus on the data from the sub‐sample of participants (*n* = 17; 12 patients, five partners) who were aware of the causal role played by HPV in their (or their partner's) cancer. These themes include: disclosing HPV to others, being diagnosed with HPV, prognosis, and questions and information about HPV.

### 3.3 Causal beliefs

This theme distinguishes between patients who knew about HPV and those that did not. It covers the cause of patients’ cancer and whether knowing the cause mattered. Almost a third of patients brought up the cause of their cancer and all others discussed it after being prompted by the researcher.

#### 3.3.1 HPV or virus

Some patients were told by their doctor that their cancer was caused by HPV (*n* = 6) or “by a virus” (*n* = 3). HPV was not always disclosed by a doctor as the cause of patients’ cancer, with a couple of patients finding out after being approached by a member of a clinical trials team asking them about taking part in research and others remained unaware of the cause. HPV was not always accepted as the cause of cancer, with one partner, after a conversation with her friend who was a nurse, dismissing HPV as the cause of her partner's cancer.

#### 3.3.2 Other and unknown cause

Some patients were not aware that HPV was the cause of their cancer despite having been tested for it. A patient whose brother‐in‐law had also been diagnosed with HPV‐OSCC specifically asked his doctor if he had HPV and was told he had “been unlucky” and that they had not found HPV. Another patient was angry that she had not been asked if she wanted a test for HPV.

A number of patients who did not recall being told what the cause of their cancer was had searched for a causal attribution; some attributed it to smoking, related it to work or genetics. Some of these patients expressed the view that they wanted to know:

I would have loved, ‘Yeah, that’s what’s caused it.’ But to this day, I don’t know what it is. (Male, 56 years, patient)

#### 3.3.3 Cause doesn't matter

Nearly half of the patients said it did not matter what had caused their cancer, the most important thing was that they “got rid of it.” The cause was also seen as something that could not be changed, “I can't go back and say I'll not do that” (Female, 66 years, patient) and that it did not affect the treatment. This partner expressed her relief that her husband's cancer was now gone:

I don’t care how it was caused. It was just that it’s gone, we hope, you know. (Female, 64 years, partner)

### 3.4 Disclosing HPV as the cause to others

A couple of patients gave examples of why they thought others did not ask them about the cause of their cancer, believing that “the cancer word's enough for most people” (Female, 61 years, patient) and “they think well you get cancer and you just get it, it's one of those things that happens” (Male, 61 years, patient). Some patients who knew that HPV was the cause of their cancer, felt uncomfortable talking about it with others: “It's not a conversation you really want to have with your daughter” (Female, 58 years, patient). One patient described telling people “It's viral, in my throat, very treatable, a type of skin cancer, it comes from HPV” and that “you don't go around broadcasting that something's sexually transmitted” (Male, 49 years, patient).

One of the younger patients said she found it easier to tell other people than her partner:

When they told me it was because of HPV I don’t think I told him for ages … I think it was easier to tell other people why as opposed to him. (Female, 41 years, patient)

One partner described how everyone assumes HNC is caused by smoking and how people reacted when she and her partner told them about HPV. Most of their friends said they looked HPV up on the internet after they had explained, as they “didn't know that could happen or exist.”

Reluctance to discuss HPV as a cause of their cancer with others was evident, not fully disclosing their diagnosis to others and viewing it as a “medical thing” so only felt comfortable talking about HPV with medical professionals. One patient explained how she felt there was “stigma attached to it [HPV],” due to experiencing negative reactions from other people and how this prevented her from disclosing the cause of her cancer. This patient felt more comfortable talking about HPV after her consultant had told her:

This is the most contagious virus in the world, 95% of people manage to disperse it through their immune system, 5% don’t and unfortunately, 5% of that 5% it turns to cancer and you’re unlucky. (Female, 42 years, patient)

Some participants also brought up Michael Douglas (a celebrity who openly described HPV and oral sex as the cause of his oral cancer) and viewed what he had said as “not helpful” and there was a “sensationalist aspect” surrounding the sexually transmitted nature of HPV. One partner described how she was happy telling people that her partner's cancer was caused by HPV before Michael Douglas said that “it's caused by oral sex.”

Well, to start with probably I was quite open telling people that [name of patient]’s cancer was caused by HPV but, after that, a bit more reluctant to discuss it. Which is a shame, because I think that more people need to be aware of this, but it’s difficult. (Female, 62 years, partner)

### 3.5 Being diagnosed with HPV

This theme describes reactions to being diagnosed with HPV and preferences for being told about HPV.

#### 3.5.1 Reactions to being diagnosed with HPV

Patients’ reactions to news of their cancer being caused by HPV were embarrassment, confusion, surprise, disappointment, shock and feeling unlucky. One patient described how she had “got a little bit of an issue still with it” and how her husband had “said something that made me feel really dirty” (Female, 42 years, patient).

One patient explained how she was embarrassed about the HPV aspect of her cancer, but how it feels less relevant now, with the cancer being most important, not the cause. Transmission of HPV to their partner was a concern for some patients and one of the partners decided to get tested privately for HPV.

#### 3.5.2 When and how to be told about HPV

There were mixed opinions from patients about when would be the best time to have been told about HPV as the cause of their cancer. Patients recognised that the initial diagnosis stage might not be the best time, but other participants thought it would have been helpful and honest to know at the beginning, when they were first told their diagnosis.

This patient also described how she was told about HPV and how this was not how she believes she should have been told:

When I found out I had it because of HPV … how it just came out totally at random … to me, if you are gonna be told something like that, which psychologically becomes very relevant, it would have probably been better to have known in a proper way not just a chat. (Female, 41 years, patient)

### 3.6 Prognosis of HPV‐related cancers

Most of the patients given information about their prognosis were told that it was treatable, curable and they could expect to recover completely. The statistics given to patients ranged from 50% to 85% survival, with research showing higher survival rates for HPV‐related diagnoses. Patients were reassured by the better prognosis given to HPV‐related diagnoses and one patient described how he focused on the words “it's treatable” (Male, 56 years, patient).

One patient interpreted 80% survival as good, but then translated this into real life and how many do not survive, when he saw the radiotherapy masks lined up in the room.

She [radiographer] said, we’ve got 42 masks at the moment. And I’ll tell you what kicked in then, there’s eight of us not going to make it then. That is what was in my mind. (Male, 53 years, patient)

### 3.7 Questions and information about their cancer

The final theme related to information patients and their partners were given about HPV, their information demands and information seeking behaviours.

#### 3.7.1 Understanding of HPV

Participants were given different information about HPV. Most participants had never heard of HPV before they were diagnosed. Those that were already aware of HPV tended to be women who had heard about it in the context of cervical cancer. Patients described discussing HPV in relation to cervical cancer and that “the HPV virus is in all of us.”

Some of the understanding about HPV that participants came away with following consultations were that it is “a virus,” is “sexually transmitted,” and “seems to affect anybody,” “you could have had it for years,” it is “the most contagious virus in the world,” “it's on the increase” and is “becoming more and more common in all ages.”

Questions participants had about HPV included whether HPV is likely to travel around their body, “what's the chance of it coming back,” “where has this [HPV] come from,” “how's it taken so long to come through,” have they still got HPV, “is it only sexually transmitted,” how long have they been carrying HPV, what is the prognosis, will the treatment get rid of HPV and are their children more at risk of HPV.

One patient was reassured by a friend who works in a hospital, that people's beliefs about HPV being down to oral sex, “is their naivety.” One patient believed that there were ways, other than oral sex, that HPV could be transmitted:

I mean surely if you can transmit the virus around, there’s going to be other ways of doing it as well, surely? It could be a cut on your hand, it could be a kiss, it could be anything, couldn’t it really? You just don’t know really. (Male, 59 years, patient)

##### Searching for information about HPV

Although the internet was a popular source of information for participants, there was an understanding that searching the internet should be done with caution. A few did not want to look for any more information on the internet because they did not want to “panic” or read the wrong information:

No. I never look on the Internet for anything like that ... it’s no disrespect to anyone who put it on there, but they don’t know like the professionals. (Male, 59 years, patient)

Some patients looking for more information found confirmation of what their doctor had told them. The internet was used to look for general information on HPV, information about the best treatment options, to research symptoms, causes, information about their doctor, explanations of tests and for further information about their particular cancer. One patient described how she “broke down” after looking up HPV on the internet as all the information said it was all because of oral sex.

For those that did further research into HPV, the level of research undertaken varied. One partner read research papers on HPV:

It seemed like the chances of recurrence were pretty low after the sort of standard treatment, which was what [name of patient] had had. So I was pretty reassured by that. (Female, 62 years, partner)

Some participants felt the information currently available was not applicable to them. One patient noted that information about HPV seems to be “centred on teenagers” and was “either aimed at young people not contracting HPV or old people not getting cancer through HPV” (Female, 41 years, patient). This particular patient felt like the information available about throat cancer was aimed at “that sort of age group—60 plus. And you are like, well, that's not me.” There were a number of participants who were satisfied with the amount of information they had been given.

##### Keeping others informed

The importance of keeping the family informed about how the patient was, was recognised, but was described by one partner as “emotionally draining” (Female, 49 years, partner). Some partners found keeping people up‐to‐date was tiring, having to answer people's texts and phone calls. One partner adopted a strategy of emailing everyone to keep them updated at once.

#### 3.7.2 Need for more research

It was acknowledged that there was a need for more research about HPV‐OSCC and that this was expected in a few years’ time. Also, that further research may lead to different and “milder treatment” for HPV cancer, with this one partner hoping this would mean people “wouldn't have had to go through the dramatic treatment he did have to go through” (Female, 62 years, partner).

#### 3.7.3 Feelings about the future

Most of the patients and their partners were positive about the future, but fear of recurrence was common among patients and their partners. This was sometimes related to HPV, such as this patient wondering “is it [cancer] more likely to come back because of this [HPV]? Is it something that stays in your body?” (Female, 61 years, patient).

## 4 DISCUSSION

This study explored the psychosocial impact of being diagnosed with HPV‐OSCC for patients and their partners. Although the medical records of all patients showed their tumour was HPV‐positive, not all patients were aware of this. This suggests a need for better information provision to ensure greater understanding of HPV in this patient group. Reactions were mixed among those who knew their cancer was caused by HPV. Some participants felt embarrassed and felt that there was a stigma associated with HPV. Other participants were not concerned about the cause of their cancer and were more interested in knowing that survival rates for a diagnosis of HPV‐OSCC were better than for non‐HPV HNCs, suggesting concerns were more cancer‐related than HPV‐related. Patients and partners who were told that HPV was the cause of their cancer had a number of questions about HPV and some sought further information. Psychosocial effects were similar to those described in previous qualitative research with HNC patients (Baxi et al., 2012; De Boer et al., 1999; Lang et al., 2013), with patients describing times of depression, anxiety and denial, but also feelings of optimism and relief.

Participants’ reactions to finding out that their HNC was caused by HPV varied. In line with findings from the cervical cancer literature, some patients reported feelings of embarrassment, confusion and concerns of transmission to their partner (McCaffery et al., 2006). Michael Douglas openly telling the media that “oral sex caused my cancer” (Shoard, 2013), was also noted to have added a “sensationalist aspect” to HPV‐OSCC. This had rendered one partner less inclined to be open about the diagnosis, while others described giving factual information about HPV being the cause when discussing with others, suggesting they were trying to avoid attaching stigma to it. Given that for some participants, the survival benefits seemed to outweigh psychosocial issues related to the sexual nature of HPV‐OSCC, this suggests that placing emphasis on survival in consultations and the media could help alleviate stigma surrounding the diagnosis of HPV‐OSCC.

One key message about HPV suggested in a previous study of health professionals (Dodd et al., 2016) was the importance of normalising HPV. Consistent with this, patients in this study reported health professionals using the link between HPV and cervical cancer to normalise HPV and emphasised telling them it is becoming more and more common. Some participants did search for more information about HPV and described how the information available was not always applicable to them and was targeted at young girls in relation to the HPV vaccination. Information sought by patients and their partners about HPV was about where HPV had come from, possible transmission and future HPV risk, supporting previous research which showed from a sample of 62 HPV‐OSCC patients, 18% sought causal information, 15% sought information about vaccinations, 10% about prevention of transmission and 10% about available treatments (Milbury, Rosenthal, El‐Naggar, & Badr, 2013). The internet was a popular source of information for participants, consistent with previous studies (Baxi et al., 2012) although patients did recognise that information on the internet is not always trustworthy and reliable. Searching for information also elicited differing reactions, with some patients finding the suggestion that oral HPV was transmitted through oral sex distressing, whereas other participants found information about the better prognosis, reassuring. Findings from a previous study with a similar population showed patients wanted more information about HPV and that “a cohesive, comprehensive, and trusted source would be valuable” (Baxi et al., 2012) (p5). These findings suggest that there is a need for information about HPV and HNC to be tailored for patients with HPV‐OSCC and developed in line with evidence‐based research. An information and support package for patients and their partners seeking information could help alleviate anxiety relating to HPV. There is a need for clear and consistent health messages aimed at diminishing stigma, fear and self‐blame (Daley et al., 2010).

Previous research in the USA has confirmed that less than half of oncologists discuss HPV with their patients (Milbury et al., 2013) and this study showed just over half of patients were aware of their HPV diagnosis. As it is becoming more recognised that patients should be informed that HPV is the cause of their cancer (Shuman & Wolf, 2010), health professionals need to decide on the best time to discuss this with patients, with patients in this study suggesting the earlier the better.

Previous research with a small sample of male HPV‐OSCC survivors also found that HPV was often overshadowed by the cancer itself and that patients were encouraged by the positive prognosis (Baxi et al., 2012). Similarly, a previous study showed that although information about HPV was seen as relevant, it was considered secondary to concerns about treatment of their cancer (Low et al., 2009).

Partners were often the source of information for others, being the ones to keep the wider family informed about the patient. Partners also sought information about HPV and other aspects of their partners’ cancer, in the effort to be as prepared as they could be, supporting previous findings from a study in New Zealand with 73 caregivers that found caregivers requested information, in an effort to improve their understanding of the situation (Richardson et al., 2015). Partners also thought about others, trying to hide their feelings from either the patient or the rest of their family. Unlike previous studies (Baxi et al., 2012; Low et al., 2009; Manne & Badr, 2008), no patients or partners in this study reported decreases in intimacy, but this may be because they were not specifically probed about their sexual relationships, but asked if there had been any changes to their relationship.

### 4.1 Strengths and limitations

This study is the first in the UK to interview patients diagnosed with HPV‐OSCC, as well as their partners. Qualitative interviews give a greater depth of data than questionnaires to provide an understanding and description of people's personal experiences. By interviewing patients and partners separately, we enabled each to share experience from their own perspective without being influenced by their partner, although it is possible that patients and their partners may have discussed the interview before participating. Interviewing both patients and their partners also allowed HPV to be discussed, which may have been too sensitive to bring up in joint interviews. Our sample of patients who were aware of their HPV status was small, so HPV was not discussed in some interviews, thereby limiting their scope. The response rate of 30% may have also limited the responses and our participants may not be representative of all patients diagnosed with HPV‐OSCC due to their recruitment from two UK hospitals. However, these two hospitals enabled us to sample from areas of different socioeconomic status and education levels and so may provide a broader representation than two hospitals in the same area of the UK. Participants were probed about changes to their relationship, but not specifically about their sexual relationships, which on reflection would have been important to discuss with this sample.

## 5 CONCLUSION

This study suggests that HPV‐OSCC has a significant psychosocial impact on patients and their partners, but that most concerns are related to dysfunction following cancer treatment and not to their HPV status. However, there are concerns and uncertainties about the sexually transmitted nature of HPV which could easily be addressed in information from a trustworthy and reliable source such as the patient's doctor. Participants in this study were ill informed and unaware regarding HPV, therefore written information for patients and their partners is also likely to be a useful resource which could be discussed around the time of diagnosis.

## CONFLICT OF INTEREST

None.
